# Use of an Elevated Avenue for Leisure-Time Physical Activity by Adults from Downtown São Paulo, Brazil

**DOI:** 10.3390/ijerph19095581

**Published:** 2022-05-04

**Authors:** Eduardo Quieroti Rodrigues, Leandro Martin Totaro Garcia, Evelyn Helena Corgosinho Ribeiro, Ligia Vizeu Barrozo, Regina Tomie Ivata Bernal, Douglas Roque Andrade, João Paulo dos Anjos Souza Barbosa, Ana Paula de Oliveira Barbosa Nunes, Rogério César Fermino, Alex Antonio Florindo

**Affiliations:** 1 Postgraduate Program in Nutrition in Public Health, Department of Nutrition, School of Public Health, University of São Paulo, São Paulo 01246-904, Brazil; joao.barbosa@ibirapuera.edu.br (J.P.d.A.S.B.); aflorind@usp.br (A.A.F.); 2 School of Arts, Sciences and Humanities, Research Group on Physical Activity Epidemiology, University of São Paulo, São Paulo 03828-000, Brazil; l.garcia@qub.ac.uk (L.M.T.G.); ehribeiro@yahoo.com.br (E.H.C.R.); douglas.andrade@usp.br (D.R.A.); ana.obnunes@hc.fm.usp.br (A.P.d.O.B.N.); 3 Centre for Public Health, Queen’s University Belfast, Belfast BT12 6BA, UK; 4 Department of Geography, School of Philosophy, Literature and Human Sciences, University of São Paulo, São Paulo 05508-080, Brazil; lija@usp.br; 5 School of Nursing, University of Minas Gerais State, Belo Horizonte 30130-100, Brazil; reginabernal@terra.com.br; 6 Research Group on Environment, Physical Activity, and Health, Postgraduate Program in Physical Education, Federal University of Technology—Paraná, Curitiba 80230-901, Brazil; rogeriofermino@utfpr.edu.br; 7 Postgraduate Program in Physical Education, Federal University of Paraná, Curitiba 80060-000, Brazil

**Keywords:** public open spaces, open streets, built environment, leisure-time physical activity, epidemiology

## Abstract

Leisure-time physical activity (LTPA) is associated with access and use of public open spaces. The President João Goulart Elevated Avenue, currently denominated Minhocão, is a facility for leisure activities that is open for people during the night and weekends. The aim of this study was to examine if the prevalence of LTPA among individuals living in the surroundings of Minhocão is different according to proximity to, and use of, the facility. We conducted a cross-sectional study with cluster sampling with people aged ≥18 years who lived in households up to 500 m, and between 501 m and 1500 m of Minhocão. The survey was conducted between December 2017 and March 2019 using an electronic questionnaire. We conducted a bivariate analysis and Poisson regression to examine possible differences in LTPA according to the proximity of residences and use of Minhocão. The analysis used post-stratification weights. A total of 12,030 telephone numbers of people were drawn (≤500 m = 6942; and >500 m to ≤1500 m = 5088). The final sample included 235 residents who returned the questionnaires. There was a higher prevalence of individuals engaging in at least 150 min per week of LTPA among users than non-users (Prevalence Ratio = 2.19, IC95% 1.66 to 2.90), independently of sex, age, education, the distance of houses to nearest accesses and number of barriers. The main five barriers related to the usage of the Minhocão were safety issues in and around the Minhocão, rainy weather, lack of vegetation, and lack of facilities. People who used the park had higher prevalence of all types of LTPA than non-users. The results can serve to inform government decision-making on the future of Minhocão.

## 1. Introduction

Previous studies have shown that engagement in leisure-time physical activity (LTPA) can prevent or attenuate a variety of chronic diseases, decrease mortality, improve quality of life and life satisfaction, and increase life expectancy [1,2,3]. However, several factors are associated with LTPA practice, including individual and contextual factors [4]. Because of this, according to Sallis et al. [5], the most promising interventions for making individuals more active should be based on an ecological model approach. This model includes the built environment and facilities for LTPA, which should be available near residences of people.

Besides the individual characteristics, physical activity (PA) is determined by factors in other levels (interpersonal, environmental, political, and global) [6]. At the environmental level, evidence shows that exposure to public open spaces may facilitate engagement in PA, especially in the leisure domain [7]. However, most studies have explored the association between parks or green areas and LTPA, which does not include the diversity of locations that can contribute to LTPA [7]. In the public open spaces context, the type, and structures may affect the population’s involvement in different types of LTPA (walking, running, and sports), which makes relevant the analysis of other spaces [8]. For example, parks and beaches may contribute to walking [9,10], while sports and recreational centers may be a better predictor of moderate to vigorous PA (MVPA) [10]. In Brazil, public open spaces usage is positively associated with age, quality of life, leisure opportunities, and satisfaction with the place [11]. Another study showed that 61% of adults who live up to 500 m from a public open spaces use the place at least once a year. Moreover, 41% use POS for PA practice [12], with a trend towards a positive association between the frequency of public open spaces usage and LTPA. Using public open spaces ≥ 4 times per week can increase in three times the probability of adults to perform at least 150 min/week of LTPA, compared with those who do not use the places [12].

From a public health perspective, public open spaces such as parks, pocket parks or squares, and bike paths are promising venues for engagement in LTPA [13,14]. Studies show that a variety of factors can influence the use of public open spaces [15,16,17,18]. The quantity and quality of these spaces, their proximity to residences, and accessibility are some factors that are associated with usage for LTPA [15,16,17,18]. In addition to public open spaces, literature has reported other environmental interventions to promote LTPA such as open streets, which are avenues or streets that are closed temporarily for cars and open for people on weekends [19,20,21]. These interventions have been referred to in the literature using different terminology according to their objectives, such as “recreational bike paths”, “open streets”, “play streets”, “leisure cycling routes”, and “leisure places” [22]. These can consist of public open spaces for recreation to provide community activities (e.g., LTPA classes) and to promote sustainable, efficient means of transport (e.g., cycling) [23].

The study by Sarmiento et al. [23] provided a summary of the available information on open streets as a public health policy in the Americas and the Caribbean drawing on different databases. The authors identified a total of 38 programs. Most interventions were conducted in Colombia, the United States, Peru, and Mexico. In addition, the authors identified some cities where initiatives entailing the closure of streets for LTPA have been implemented, including São Paulo, in Brazil. The problem is the lack of studies evaluating the effectiveness of these interventions in increasing PA in adults living in low- and middle-income countries. Evidence shows that open streets provide opportunities for many PA types, such cycling, walking, running, and skating [24]. The evaluation of program effectiveness shows that 60% of participants of an open street accumulated enough LTPA to meet the recommended PA levels for health, demonstrating the program’s cost-effectiveness from a public health perspective [25,26].

The “President João Goulart Elevated Avenue”, currently denominated Minhocão (or “big earthworm” in English, because of the shape of the 3.4 km avenue), was listed in the study by Sarmiento et al. [23] as an open street. This elevated avenue was inaugurated in 1971 only to ease traffic congestion for motor-vehicle traffic [27,28]. However, this facility started to cause many problems for local residents such as excess noise, dirt, and air pollution [27]. Because of this, São Paulo City Hall authorities have closed this elevated avenue to the motor-vehicle traffic initially at nights and later also on weekends in an effort to ameliorate these problems [28]. Since this initiative was taken, the local population has been using voluntarily the elevated avenue for different LTPA, such as walking, running, and biking at nighttime and on weekends. In 2018, the municipal councilors of São Paulo City approved the Minhocão Park law [29], whose project enabled the discussion for gradual phasing out of use of the elevated avenue by motor vehicles. The project was included in the New Master Plan of São Paulo City in 2014 [30]. However, the discussion also includes the possible demolition of the elevated avenue and in 2021 the Minhocão Park law was annulated.

To better inform the discussions about the future of the Minhocão, it is important to verify potential contributions of this elevated avenue to LTPA of people living near it. The rationale behind this study is underpinned by scientific and policy evidence. Firstly, a study conducted by Florindo et al. [7] showed that adults who lived in São Paulo City between 2014 and 2015 and that had access to two or more public open spaces (such as parks, squares, or bike paths) within a 500 m radius from their homes had significantly higher likelihood to engage in leisure-time walking compared with those without these spaces available in their neighborhood. The study was conducted with adults in Curitiba in south Brazil; it showed that users of parks at least once a week had a higher prevalence of leisure-time walking than non-users [31]. However, there are few studies that evaluated the contributions of open streets such as Minhocão to LTPA in low- and medium-income countries. In 2019, Nogueira et al. [32] conducted an exploratory study with 265 adult users of the Minhocão to describe some demographic, social, and environmental characteristics. The main results showed that most users walk or cycle to the place (95%), lived less than 1000 m from Minhocão (76%), and performed LTPA (60%).

Lastly, from a political standpoint, discussions on the future of the elevated avenue (demolition or creation of a park) gained impetus in 2021 with the review of the New Master Plan for São Paulo approved in 2014 [30]. Thus, the results of the present study can serve to foster this discussion. Therefore, the objective of the present study was to examine if the prevalence of LTPA among individuals living in the surrounding of Minhocão is different according to proximity of their residences and according to the usage of the facility.

## 2. Materials and Methods

### 2.1. Study Site

São Paulo is the most populous city in Brazil and the eighth in the world, with an estimated population in 2021 of 12,396,372 million inhabitants living in 1,521,110 km^2^ [33]. The city concentrates 11% of the Gross Domestic Product of Brazil. Currently, it has 651.9 km of cycle paths and cycle lanes [34], 101.1 km of subway lines with 89 stations [35], and 106 parks.

**Figure 1:**
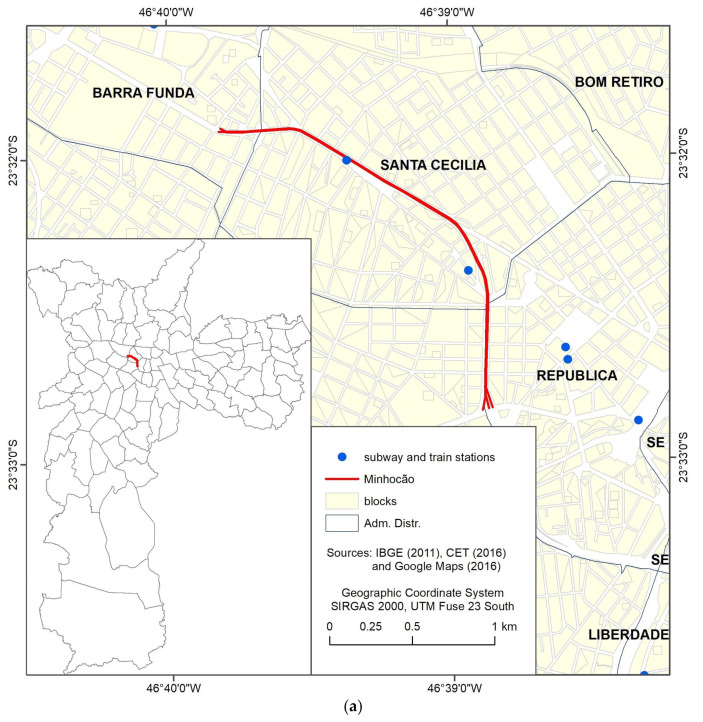

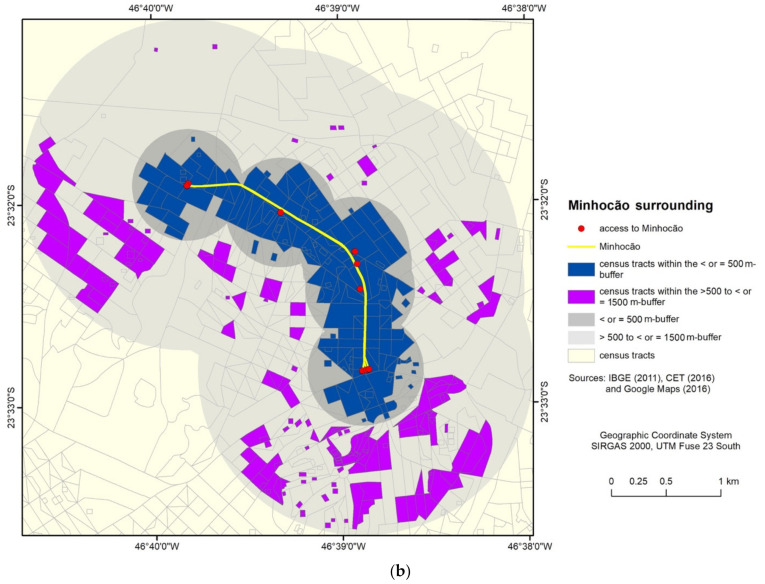
Location of Minhocão in São Paulo City, Brazil (**a**), and census tracts of residents randomly selected according to buffer zones with different radiuses from entrances to Minhocão (**b**).

The Minhocão is an elevated highway (Figure 1a) with 3.4 km extension, five meters above the ground, dual roadway, with four lanes in each direction separated by a central concrete barrier wall. The elevated avenue has nine points of access. Some of its sections are only five meters away of the facades of buildings on its sides [36]. The elevated highway is closed for motor-vehicle traffic and open for recreational activities, including physical activity, from Monday to Thursday from 8 p.m. to 7 a.m. and on weekends from 8 p.m. on Friday until 6 a.m. on Monday.

### 2.2. Study Design and Sample

We conducted a cross-sectional telephone and email survey. The target population comprised individuals ≥ 18 years of age who lived in households within 1500 m of an access to Minhocão (Figure 1b).

The sample size was calculated considering a confidence level of 95%, test power of 80%, proportion of exposed positives (i.e., people living up to 500 m an access and who practiced ≥150 min per week of LTPA) of 45%, and non-exposed positives (i.e., people living between 501 and 1500 m of an access and who practiced ≥150 min per week of LTPA) of 34%. The proportions were obtained from a study conducted by Pazin et al. [37]. The required sample size was 616 adults in total (308 adults living up to 500 m of an access, and 308 adults living between 501 and 1500 m of an access).

Households located within 1500 m of an elevated highway access point were randomly selected. Using ESRI ArcMap version 10.3 (Redlands, CA, USA), we identified the census tracts lying fully within one of the two buffer zones of interest: ≤500 m (Group 1), and >500 to ≤1500 m radius (Group 2) from a highway access (Figure 1b). Census tracts were based on the 2010 national census conducted by the Brazilian Institute of Geography and Statistics. Buffer sizes were based on the results from the study by Pazin et al. [37]. We identified 164 tracts in Group 1 and 164 other tracts in Group 2.

We conducted cluster sampling with a two-stage selection process and probability proportional to the cluster size. The census tracts were the primary sampling units. Fifty-eight tracts were randomly selected in Group 1 and 37 tracts in Group 2. The second sampling units were the addresses (street name, number, and postal code of each randomized tract), with a total of 15 addresses randomly selected per tract. After random selection of addresses, we randomly selected home telephone numbers. The list of telephone numbers associated to a given address was retrieved from the websites of the telephone service operators. All available telephone numbers associated with the selected addresses were collected. For example, if an address corresponded to an apartment building, all available telephone numbers of all apartments were selected. A total of 12,030 telephone numbers of individuals living in the vicinity of Minhocão (≤500 m = 6942; and >500 m to ≤1500 m = 5088) were randomly selected.

### 2.3. Telephone Call to Sampled Participants

Undergraduate students from the Physical Activity Epidemiology Group at University of São Paulo were trained for a total of 15 h on conducting the telephone calls. The students were provided a password-protected spreadsheet containing all the randomly selected telephone numbers. In the event of three unsuccessful attempts to the same number at different times and on different days, no further calls were made to the number. In the event of successful calls, interviewers explained the objectives and importance of the survey and invited all adults (≥18 years of age) members of the household to take part in the study. All individuals who agreed to participate were asked to provide an e-mail where the electronic survey questionnaire could be sent to. Follow-up letters and email messages were sent to survey participants to stimulate response rate. On average, 1.3 call was needed per number, resulting in successful contact for 6977 telephone numbers (58%). We posted follow-up letters to 1306 households with at least one person who agreed on taking part in the survey.

### 2.4. Electronic Survey Questionnaire

The survey was conducted between December 2017 and March 2019. The questionnaire comprised of open and multiple-choice questions divided into six blocks: 1. sociodemographic variables, self-rated health, and self-reported weight and height; 2. LTPA; 3. use of Minhocão for LTPA; 4. personal and environmental barriers to use the Minhocão; 5. social support; and 6. self-efficacy. For this paper, responses to blocks 1, 2, 3, and 4 were analyzed.

LTPA was assessed using the long version of the International Physical Activity Questionnaire (IPAQ), which was validated in a sample of Brazilian adults for its applicability in electronic form [38] and via e-mail [39], demonstrating good validity, reproducibility, and concordance in both studies. This version of the IPAQ was standardized to collect information on engagement in LTPA during the past 7 days in minutes per day and number of days per week spent on walking, moderate, and vigorous intensity activities. Participants were also probed about whether they used the Minhocão for LTPA (yes or no) in the last 7 days.

Questions about environmental barriers to use Minhocão were based on the scale proposed and validated by Reis, Nascimento, and Petroski [40], which assesses factors that inhibit or encourage the practice of PA in urban parks. Study participants answered the following question: “What are the main environmental barriers for you to not practice physical activity in your free time at Minhocão?”. Participants answered yes, no, do not know, or do not want to inform for 15 items: (1) rainy weather; (2) cold weather; (3) high temperatures; (4) air pollution; (5) noise pollution; (6) park aesthetics; (7) lack of facilities (such as bathrooms and drinking fountains); (8) lack of exercise equipment (such as benches, bars and backrests); (9) lack of vegetation (such as trees, plants and green areas); (10) lack of lighting; (11) safety issues in the park; (12) safety issues in the vicinity of the park; (13) number of accesses to the park; (14) distance between park accesses; (15) and distance between residence and the park. The sum of barriers was calculated and categorized into tertiles.

The questionnaire was developed on the free platform Google Forms, allowing form sharing via any communication media using a URL link to the questionnaire and real-time storage of responses on the cloud in the form of spreadsheets, with no need for manual data entry.

### 2.5. Data Analyses

Means, standard deviation (sd), and absolute (*n*) and relative frequencies (%), and confidence intervals of 95% (CI 95%) were used to describe the data.

The chi-squared test was applied to explore associations between (a) distance from the participants’ households to the closest entrance to the elevated avenue (≤500 m; and >500 m to ≤1500 m); and (b) usage of the Minhocão (yes or no) and leisure-time walking, moderate and vigorous LTPA. In cases where chi-squared assumptions were not met (cells containing expected values ≥ 5), then Fisher’s exact test was employed.

For associations with *p*-value ≤ 0.05 in the bivariate analyses, we used Poisson regression models to determine the direction and magnitude of association between different types of LTPA. Multivariate models were run in four phases: 1. without adjustments; 2. adjusted for sex, age, and education; 3. model 2 plus distance to closest entrance (≤500 m, and >500 m to ≤1500 m); and 4. model 3 plus number of environmental barriers. The prevalence ratio (PR) was calculated with CI95%. Post-stratification weights were used to correct potential biases due to low response rate. Weights were calculated using the rake method. The age and sex of the population from the 2010 Census were used as a reference population in the construction of the weights. All statistical analyses were performed in Stata, version SE 16.1 (StataCorp, College Station, TX, USA) with post-stratification weights using the survey module (svy) [41].

## 3. Results

Of the 6977 telephone numbers successfully contacted, 421 email addresses of people interested in participating in the survey were obtained. Of those, 242 questionnaires were completed. Six questionnaires were subsequently excluded for containing non-randomized addresses, while one questionnaire was not included because the respondent was under 18 years of age. A total of 235 residents living near Minhocão were included in the analysis.

Respondents were predominantly male (53.6%), with 18 to 29 years of age (28.9%) (mean = 42.4 years, sd = 1.2 year), started or completed an undergraduate course (56.2%), and lived within 500 m of park entrances (66%). Only 34% used the facility for LTPA. Most of the sample reported walking and moderate LTPA (Table 1).

The main five barriers related to the usage of the Minhocão were, respectively, safety issues in and around Minhocão, rainy weather, lack of vegetation, and lack of facilities (Table 2). Interestingly, accesses to the Minhocão in general was a barrier to less than 25% of the respondents. The mean of number of barriers reported was 6.9 (sd = 0.27).

LTPA in the past week was associated with proximity to the nearest access to Minhocão (Table 3).

Minhocão usage was positively associated with walking, moderate, and vigorous LTPA (Table 4).

There was a higher prevalence of individuals engaging in leisure-time walking, moderate and vigorous activities, and total LTPA among Minhocão users than non-users, independently of sex, age, and education, of the distance from their houses to the nearest access, and of barriers related to the usage of Minhocão (Table 5).

## 4. Discussion

This study examined if the prevalence of LTPA among individuals living in the surroundings of Minhocão is different according to proximity to, and use of, the facility, an elevated avenue that is transformed into an open street for people during the nights and on the weekends. We found that Minhocão users engaged in more LTPA than non-users. People that used the facility had a higher prevalence of all types of LTPA investigated, independently of the distance between their houses and the nearest access, and environmental barriers. However, proximity to the nearest access of the elevated avenue was not associated with LTPA.

We did not observe large differences in the proportion of people by sex and age groups. However, most of the people interviewed had higher education. The results about social and demographic characteristics were similar to those found by Nogueira et al. [32], who interviewed 265 adults users of Minhocão in 2019. 

The LTPA level was similar to the results found by Nogueira et al. [32]. However, LTPA prevalence was twice the results found by the telephone surveillance system of the Ministry of Health that evaluated a representative sample of 2052 adults living in São Paulo City in 2019 [42]. In addition, prevalence was higher than that observed in a household survey conducted with a representative sample of 3145 adults from São Paulo City in 2014 and 2015 [14]. These results might indicate that people living near Minhocão are more physically active in the leisure domain.

Most of the people lived up to 500 m from an access to Minhocão; however, most of the respondents did not use the Minhocão. Nogueira et al. [32] showed that 47% of adult users interviewed in 2019 lived up to 500 m from there, 76.5% up to 1 km, and 80% up to 1.5 km. That means that most users lived up to 1.5 km from the Minhocão, which was the maximum distance we used to select the sample of this study.

The main environmental barriers related to the use of the Minhocão were related with safety. Nogueira et al. [32] identified that most people went to Minhocão by walking, spending up to 10 min to reach it. The Minhocão is open for people during the night and weekend days. Safety issues are important because the region where the elevated avenue is has a high index of criminality, particularly thefts and robberies [43], and safety perception is associated with walking for transportation [44]. In addition, the absence of facilities (such as restrooms and drinking fountains) and vegetation were important barriers. A study conducted with adults from Perth, Australia, showed that the quality of public open spaces, including the presence of green areas such as gardens and facilities such as water fountains and restrooms, was associated with a higher likelihood of LTPA practice [45].

Studies have shown that facilities and open streets such as Minhocão are important to promote LTPA [7,15,22,23,46,47], particularly in a megalopolis such as São Paulo, where environmental inequities are a big problem [30]. Few studies have examined the relationship between public open spaces usage and LTPA, especially in higher intensity activities [12,48,49,50,51]. In general, positive and significant associations have shown that using these places can increase leisure walking between 17 and 390%, and MVPA between 55 and 420% [49,50]. The wide variability of the association magnitude can be explained by a countries’ contextual characteristics and public open spaces settings, as well as residual confounding bias. For instance, Mackenbach et al. [49] analyzed the usage of formal recreational facilities (public and private courts, parks, and gyms) among European adults and reported an increase of 17% and 55% in walking and MVPA during leisure time, respectively. On the other hand, Salvo et al. [50] examined the usage of formal and informal public open spaces by adults from three Latin American countries (Brazil, Colombia, and Mexico) and reported an increase of 4.9 and 5.2 times in the odds of walking and MVPA, respectively. An essential contextual characteristic for the latter study was the high usage of informal public open spaces in Colombia, such as shopping malls (54%) [50]. The differences mentioned above reinforce the relevance of analyzing different locations, contexts, and realities when examining the possible impact of public open spaces usage on LTPA.

The results of this paper were similar to those observed by a cross-sectional study of 749 intentionally selected adults in four parks (*n* = 303) and four squares (*n* = 446) in Curitiba city, southeast Brazil, which showed that users of parks at least once a week had a higher prevalence of leisure-time walking (PR = 1.30; CI95% 1.03 to 1.64) than non-users [31].

The most innovative aspect of this study was to show that the association between usage of Minhocão for LTPA and LTPA levels was statistically independent of the distance between houses and the nearest access to Minhocão and environmental barriers. Moreover, we did not find a statistical association between proximity to the nearest accesses to Minhocão and LTPA practice. Nogueira et al. [32] also found similar results and did not find a significant statistical association between the distance people live from Minhocão and LTPA. This result is different from other studies that showed that distance between people’s houses and facilities such as public open spaces was associated with LTPA [14,37,52].

The study conducted by Florindo et al. [7] with a sample of 3145 adults of São Paulo City, Brazil, showed that individuals who had access to two or more public open spaces (such as parks, squares, or bike paths) within a 500 m radius of their homes had a significantly greater likelihood of engaging in any level of leisure-time walking (OR = 1.65; CI95% 1.09 to 2.55) compared with individuals without access to these spaces near their homes. However, results were not statistically significant for greater distances. A cross-sectional study carried out in Hong Kong between 2007 and 2008 reported similar results, showing that the presence of parks within a 400 m radius of residences of older adults was associated with engagement in leisure-time walking (OR = 1.03, 95%CI 1.02 to 1.05) [52]. A natural experiment conducted with 519 adults from Florianopolis city, Brazil, showed that after 2.5 years of building a new walking and cycling route, only people who lived up to 500 m from this facility had a statistically significant increase in minutes of leisure-time walking [37].

The present study has three main limitations. The first is that the online surveys were only completed by respondents who had a landline, an email address, and internet access via computer, smartphone, or tablet. With advances in technology, many telephone users rely on mobile phones and no longer have a landline. However, according to the National Telecommunications Agency, 89.3% of households had landlines in São Paulo State in 2019 [53] and, according to Bernal et al. [54], the use of domestic landlines is recommended for carrying out epidemiological surveys in areas whose coverage exceeds 70%. The use of post-stratification weights by age and sex according to the Population Census helped decrease this limitation. 

Another aspect to note was the low response rate and the self-selection bias. Individuals may have declined to answer the questionnaire because they had limited accessibility to the internet or familiarity with computers, smartphones, emails, and the internet, particularly among low socioeconomic level families and older people. In addition, it is unclear whether the high refusal rates reflected negative views about the park implementation among local residents, or whether most respondents were in favor of creating the park. This sample size might affect the power of our analysis examining the relationship between the proximity of residences to the nearest park entrance and LTPA. The use of post-stratification weights in the analysis diminishes the negative impact of the low response rate on the power of our analyses, although it might not fully correct self-selection bias.

Finally, the interpretation of our results is limited by the cross-sectional design of the study. For instance, we do not know the percentage of people living in the surroundings of the Minhocão who practiced LTPA before it was opened for recreational activities, or how many people moved near the Minhocão because it is an open public space for LTPA. This prevents us from reaching any conclusions about the effect of the Minhocão on people’s LTPA levels.

## 5. Conclusions

This study showed that the users of Minhocão are more physically active in their leisure time than non-users. However, the proximity between people’s residences and the nearest access to the elevated avenue was not statically associated with LTPA. We believe that these results can inform new discussions about the elevated avenue and inform the government and population during decision-making about the future of this open public space.

Despite the limitations of our study, we believe that the results showing that the users of Minhocão are more physically active in LTPA than non-users are important to support this open public space for physical activity promotion in downtown São Paulo City. The results are relevant to help close the gap of evaluation studies of open public spaces for physical activity promotion in low- and middle-income countries. For example, in Colombia, a free community program called “Ciclovía Recreativa” makes available 121 km of temporarily pedestrianized streets to the public for LTPA on pre-defined days and hours [23]. A cross-sectional study found that 60% of program users accumulate enough LTPA to meet the recommended levels of overall physical activity [25].

Finally, actions and programs coordinated by City Hall authorities that exploit the built environment creating open streets for people during the weekends, as it is performed for Minhocão, are important population-level interventions with the potential to reach a large number of people, contributing to the World Health Organization Global Action Plan for physical activity promotion [55]. These efforts are important to continue with good results in public health such as the increase of 7.8% in LTPA levels among adults residing in São Paulo between 2006 and 2016 [56]. Such initiatives are especially relevant amid the COVID-19 pandemic, where recent Brazilian epidemiological studies have revealed a decline in LTPA among adults [57].

## Figures and Tables

**Table 1:** Social and demographic characteristics, usage of the Minhocão, and leisure-time physical activity of respondents residing up to 1500 m from an access to Minhocão. São Paulo City, São Paulo State, Brazil, 2017–2019.

**Variables**	% *	(CI95%)
**Sex**		
Female	46.4	(39.4–53.6)
Male	53.6	(46.4–60.6)
**Age Group (years)**		
18–29	28.9	(22.1–36.8)
30–39	21.5	(16.2–27.9)
40–49	16.3	(11.7–22.2)
50–59	14.2	(10.9–18.3)
≥60	19.1	(14.6–24.8)
**Education**		
Up to complete high school	15.1	(10.4–21.4)
Incomplete or complete undergraduate	56.2	(49.0–63.2)
Incomplete or complete postgraduate	28.7	(22.8–35.4)
**Distance of Residence to an Access to Minhocão**		
≤500 m	66.8	(59.8–73.3)
>500 to ≤1500 m	33.2	(26.8–40.2)
**Use of Minhocão for LTPA**		
No	65.7	(58.5–72.3)
Yes	34.3	(27.7–41.6)
**Leisure-Time Walking**		
No	42.1	(35.3–49.3)
Yes	57.9	(50.7–64.7)
**Moderate LTPA**		
No	43.7	(36.8–50.9)
Yes	56.3	(49.1–63.2)
**Vigorous LTPA**		
No	66.8	(59.4–73.5)
Yes	33.2	(26.6–40.6)
Total LTPA in the Week **		
<150 min	45.3	(38.2–52.5)
≥150 min	54.7	(47.5–61.7)

Notes: * post-stratification weight by age and sex; ** leisure-time physical activity (LTPA) including walking, moderate and vigorous activities. Abbreviations: CI: confidence interval. LTPA: leisure-time physical activity.

**Table 2:** Environmental barriers to leisure-time physical activity practice by residents around the Minhocão. São Paulo–SP, 2017–2019.

Variables	% *	(CI95%)
**Rainy Weather**		
No	28.3	(22.1–35.5)
Yes	71.7	(64.5–77.9)
**Cold Weather**		
No	64.6	(57.3–71.2)
Yes	35.4	(28.8–42.7)
**High Temperatures**		
No	57.0	(49.6–64.2)
Yes	43.0	(35.9–50.4)
**Air Pollution**		
No	57.7	(50.2–64.9)
Yes	42.3	(35.1–49.8)
**Noise Pollution**		
No	70.8	(63.7–77.1)
Yes	29.2	(22.9–36.3)
**Park Aesthetics**		
No	66.5	(59.1–73.2)
Yes	33.5	(26.8–40.9)
**Lack of Facilities in the Park**		
No	42.0	(34.7–49.6)
Yes	58.0	(50.4–65.3)
**Lack of Exercise Equipment in the Park**		
No	49.6	(42.2–57.1)
Yes	50.3	(42.9–57.8)
**Lack of Vegetation in the Park**		
No	37.1	(30.2–44.5)
Yes	62.9	(55.5–69.8)
**Lack of Lighting in the Park**		
No	42.2	(34.9–49.7)
Yes	57.8	(50.3–65.1)
**Safety Issues in the Park**		
No	22.0	(16.4–28.9)
Yes	78.0	(71.2–83.6)
**Safety Issues around the Park**		
No	24.5	(18.5–31.6)
Yes	75.5	(68.5–81.5)
**Number of Park Accesses**		
No	75.6	(68.5–81.5)
Yes	24.4	(18.5–31.5)
**Distance between Park Accesses**		
No	77.7	(70.8–83.3)
Yes	22.3	(16.7–29.2)
**Distance between Homes and the Park**		
No	84.5	(78.3–89.2)
Yes	15.5	(10.9–21.7)

Notes: * post-stratification weight by age and sex. Abbreviation: CI: confidence interval.

**Table 3:** Leisure-time physical activity among residents in the surroundings of Minhocão, according to distance to nearest access to Minhocão. São Paulo City, São Paulo State, Brazil, 2017−2019.

LTPA in Past Week	≤500 m		501 to ≤1500 m		
	% *	(95%CI)	% *	(95%CI)	p **
**Walking**					
No	40.3	(32.1–49.1)	45.8	(33.9–58.2)	0.470
Yes	59.7	(51.0–67.9)	54.2	(41.8–66.1)	
**Moderate**					
No	43.1	(34.7–51.9)	45.0	(33.2–57.4)	0.803
Yes	56.9	(48.1–65.3)	55.0	(42.6–66.8)	
**Vigorous**					
No	64.3	(55.1–72.5)	71.8	(58.8–81.9)	0.327
Yes	35.7	(27.5–44.9)	28.2	(18.1–41.2)	
Total LTPA ***					
<150 min per week	44.8	(36.3–53.6)	46.3	(34.4–58.7)	0.844
≥150 min per week	55.2	(46.4–63.7)	53.7	(41.3–65.6)	

Notes: * post-stratification weight by age and sex; ** *p*-values for Pearson’s chi-squared test; *** leisure-time physical activity (LTPA) including walking, moderate or vigorous activities. Abbreviations: CI: confidence interval; LTPA: leisure-time physical activity.

**Table 4:** Association between use of Minhocão and leisure-time physical activity among adults living in the surroundings of Minhocão. São Paulo City, São Paulo State, Brazil, 2017−2019.

LTPA in Past Week	Use of Minhocão for LTPA				
	No		Yes		
	% *	(95%CI)	% *	(95%CI)	p **
**Walking**					
No	60.5	(51.4–68.9)	13.1	(6.3–25.1)	<0.0001
Yes	39.5	(31.1–48.6)	86.9	(74.9–93.7)	
**Moderate**					
No	61.3	(52.2–69.7)	13.3	(6.8–24.4)	<0.0001
Yes	38.7	(30.3–47.8)	86.7	(75.6–93.2)	
**Vigorous**					
No	76.6	(67.6–83.8)	44.1	(31.9–57.0)	<0.0001
Yes	23.4	(16.2–32.4)	55.9	(43.0–68.1)	
Total LTPA***					
<150 min per week	63.1	(53.9–71.4)	11.7	(5.8–21.9)	<0.0001
≥150 min per week	36.9	(28.6–46.1)	88.3	(78.1–94.2)	

Notes: * post-stratification weight by age and sex; ** *p*-values for Pearson’s chi-squared test; *** Leisure-time physical activity (LTPA) including walking, moderate or vigorous activities. Abbreviations: CI: confidence interval; LTPA: leisure-time physical activity.

**Table 5:** Prevalence ratios of leisure-time physical activity according to use of Minhocão among adults living in the surroundings of Minhocão. São Paulo City, São Paulo State, Brazil, 2017–2019.

Use of Minhocão for LTPA	Model 1 PR * (95%CI)	Model 2 PR * (95%CI) **	Model 3 PR * (95%CI) ***	Model 4 PR * (95%CI) ****
**Walking**				
No	1	1	1	1
Yes	2.03 (1.62–2.56)	1.95 (1.55–2.47)	1.95 (1.54–2.46)	2.28 (1.74–2.98)
**Moderate**				
No	1	1	1	1
Yes	2.18 (1.72–2.77)	2.15 (1.68–2.74)	2.15 (1.68–2.74)	2.12 (1.63–2.76)
**Vigorous**				
No	1	1	1	1
Yes	2.50 (1.64– 3.82)	2.25 (1.46–3.46)	2.22 (1.44–3.43)	2.00 (1.29–3.11)
Total LTPA *****				
<150 min per week	1	1	1	1
≥150 min per week	2.38 (1.86–3.04)	2.25 (1.75–2.90)	2.25 (1.75–2.90)	2.19 (1.66–2.90)

Notes: * post-stratification weight by age and sex; ** adjusted for sex, age and education; *** adjusted for sex, age, education and distance to nearest access; **** adjusted for sex, age, education, distance to nearest access and number of barriers; ***** leisure-time physical activity (LTPA) including walking, moderate or vigorous activities. Abbreviations: PR: prevalence ratio; CI: confidence interval; LTPA: leisure-time physical activity.

## Data Availability

The data presented in this study are available on request from the corresponding author.
